# Impact of COVID-19 on quality of life in Peruvian older adults: construct validity, reliability and invariance of the COV19—Impact on Quality of Life (COV19-QoL) measurement

**DOI:** 10.1186/s41155-023-00256-0

**Published:** 2023-05-22

**Authors:** Tomás Caycho-Rodríguez, Carlos Carbajal-León, Lindsey W. Vilca, Mario Reyes-Bossio, Miguel Gallegos, Renzo Carranza Esteban, Martin Noe-Grijalva, Walter L. Arias Gallegos, Mariel Delgado-Campusano, Águeda Muñoz-del-Carpio-Toia

**Affiliations:** 1 Facultad de Psicología, Universidad Científica del Sur, Campus Villa II, Ctra. Panamericana S 19, Villa EL Salvador, Lima, Peru; 2 South American Center for Education and Research in Public Health, Universidad Norbert Wiener, Lima, Peru; 3 Facultad de Psicología, Universidad Peruana de Ciencias Aplicadas, Lima, Peru; 4 Programa de Pós-Graduação em Psicologia, Pontificia Universidade Católica de Minas Gerais, State of Minas Gerais Belo Horizonte, Brazil; 5 Centro Interdisciplinario de Investigaciones en Ciencias de la Salud y del Comportamiento. Consejo Nacional de Investigaciones Científicas y Técnicas, Entre Ríos, Argentina; 6 Facultad de Ciencias de La Salud, Grupo de Investigación Avances en Investigación Psicológica, Universidad San Ignacio de Loyola, Lima, Perú; 7 Escuela de Psicología, Universidad César Vallejo, Trujillo, Perú; 8 Departamento de Psicología, Universidad Católica San Pablo, Arequipa, Perú; 9 Escuela de Medicina Humana, Universidad Católica de Santa María, Arequipa, Perú

**Keywords:** Older adults, Quality of life, COV19-QoL, Validity

## Abstract

The aim of the present study was to translate into Spanish and evaluate the psychometric evidence of the Impact on Quality of Life (COV19-QoL) applied to a sample of Peruvian older adults (*N* = 298; 58.1% women, 41.9% men, mean age 65.34 years [SD = 11.33]). The study used techniques from the Classical Test Theory (CTT) and Item Response Theory (IRT). The findings confirmed the single factor structure of the COV19-QoL, high internal consistency reliability, measurement invariance by gender, and all items demonstrated adequate discrimination and difficulty indices. In this sense, the items allow adequate discrimination between low, medium and high levels of the impact of the COVID-19 pandemic on quality of life. In addition, a greater perceived impact of the pandemic on quality of life is necessary to answer the higher response options of the COV19-QoL. In conclusion, the COV19-QoL is a valid measurement scale of the impact of the COVID-19 pandemic on the quality of life of Peruvian older adults.

## Introduction

In 2019, there were 1 billion adults over the age of 60 in the world; while by 2030, one in six people will be 60 or older, and by 2050, it is projected that the older adult population will reach 2.1 billion (United Nations, 2019). Peru is a Latin American country with a rapidly aging population (López, 2022). According to the National Institute of Statistics and Informatics (Instituto Nacional de Estadística e Informática [INEI], 2016), in 2020, the population of people over 60 years of age was 3,593,054; while it is estimated that by 2025 and 2050, this population will rise to 4,309,593 and 8,738,032, respectively. These figures, plus the appearance of the COVID-19, aggravate the problem of older adults and create a set of challenges for public policy makers (López, 2022). Older adults were one of the population groups most affected by the pandemic, not only because of their higher risk of mortality from COVID-19, but also because of the consequences for physical and mental health due to the restrictions imposed by the different governments to mitigate the spread of the disease (Herrera et al., 2021). It was estimated that the mortality risk for people aged 60 years was 3.6%, a percentage that increased to 8 and 14.8% for people between 70 and 80 years respectively (Brooke, & Jackson, 2020). Approximately 80% of deaths related to COVID-19 have been in older adults (Bidzan-Bluma et al., 2020); while, in Peru, 69.80% of deaths due to COVID-19 were in people older than 60 years of age (Ministerio de Salud [MINSA], 2022).

In response to the COVID-19 health emergency, on March 15, 2020, the Peruvian government declared a state of national emergency due to the outbreak of COVID-19, establishing mandatory social immobilization, restrictions on commercial, cultural, sporting and recreational activities, temporary closure of borders and reduction of land and air transportation, restriction of various rights such as freedom, personal safety and inviolability of the home, mandatory use of masks to circulate on public roads and in indoor places, as well as social distancing, frequent hand washing, among others (Fhon et al., 2022). The Peruvian government considered older adults as a vulnerable population to COVID-19 (especially older adults with chronic diseases such as diabetes, arterial hypertension, cardiovascular diseases, etc.) and ordered this group to adhere to mandatory home immobilization, even after the restrictive measures were lifted. In this regard, it is important to consider that, in Peru, about 42% and 29.5% of nuclear and extended families, respectively, had an elderly person among their members; therefore, they could have been exposed to possible cases (Mendoza-Saldaña & Viton-Rubio, 2022).

Home confinement for the elderly was from mid-March through November 2020, with some exceptions such as in the case of medical emergencies or the need to obtain food or meet basic needs. However, complying with this measure was difficult, since 14.9% of the elderly are in poverty, 53.5% are self-employed and 63% do not have a retirement pension (Mendoza-Saldaña & Viton-Rubio, 2022). These characteristics force many older adults to venture out into the streets to work and take up informal jobs, creating a greater exposure to the risk of COVID-19 infection. It was not until mid-October 2020 that older adults were allowed to take 60-min walks outdoors three times a week. Many of the strategies and precautionary measures implemented by the Peruvian government to mitigate the spread of COVID-19 were given through different media. However, about 66.3% and 61.1% of households with at least one senior citizen among their members do not have Internet and television service, respectively; while approximately 17% of older adults nationwide are illiterate (INEI, 2020). This may have made access to preventive information more difficult for Peru's older adult population. Following the recommendation to wash hands frequently was also a problem for a significant group of the older adult population, as 10% of households with at least one older adult member did not have access to a safe water supply (INEI, 2020).

Measures to control COVID-19 have had a negative effect on quality of life and have contributed to an increase in the presence of anxiety symptoms and depression (Ferreira et al., 2021). In Peru, it has been reported that after the declaration of a health emergency in the country, a group of older adults living in shelters reported a decrease in their will to live and satisfaction with their health, as well as an increase in anxiety symptoms (Caycho-Rodríguez et al., 2021). Likewise, the pandemic has increased fear and anxiety about COVID-19, which has generated higher levels of anxiety and depression in older Peruvians (Caycho-Rodríguez et al., 2022a, 2022b, 2022c). Another study indicated that older adults suffering from obesity, respiratory diseases, fatigue and hearing problems reported a lower quality of life; in addition, having osteoarticular diseases and fatigue generated mobility difficulties to perform daily activities (Tenorio-Mucha et al., 2021). Finally, it has been reported that approximately 39% of older adults presented depressive symptoms, either moderate (28.3%) or severe (10.7%); while being 76 years of age or older, not engaging in recreational activities and having comorbidities were significantly associated with the presence of depressive symptoms (Sáenz et al., 2021).

Worldwide, it has been reported that about 37.1% of older adults reported symptoms of anxiety and depression (Meng et al., 2020); while, more than three-fourths of older adults reported not having good mental health during the pandemic (Negarestani et al., 2021). In addition, sleep disturbances and a reduction in physical activity have been observed (Lebrasseur et al., 2021). Similarly, it has been reported that older adults have reported a deterioration in their quality of life compared to the year prior to the pandemic (Siette et al., 2021). It should be noted that it has been suggested that psychological problems, including symptoms of depression and anxiety, as well as impaired quality of life resulting from COVID-19, may persist for many years after the pandemic (Siette et al., 2021). However, it has also been suggested that advanced age may be a factor that mitigates the impact of COVID-19 on quality of life due to its ability to adapt to adversity (Parlapani et al., 2021).

Given this scenario, and from a public health perspective, it is important to evaluate the impact of the pandemic on the quality of life of older adults. This is even more important due to the vulnerability of this age group and the consideration that Peru was one of the countries that had one of the strictest quarantines during the pandemic (Tenorio-Mucha et al., 2021). During the pandemic, different studies have used a variety of instruments to measure quality of life, such as the Brunnsviken brief quality of life scale (BBQ; Lindner et al., 2016), World Health Organization Quality of Life-brief version (WHOQOL-BREF; Power et al., 2005). In Spanish, the Perceived Quality of Life Scale (Escala de Calidad de vida Percibida) has been used to measure the quality of life (Verdugo et al., 2009), the EuroQol-5D (Balestroni & Bertolotti, 2012) and the Multicultural Quality of Life Index (MQLI; Jatuff et al., 2007), among others. However, none of the above measures have specifically assessed the impact of the pandemic on quality of life. Instruments that measure general aspects of quality of life and mental health generate under- or over-diagnosis (Ransing et al., 2020). In addition, these instruments have measured aspects of quality of life in the early phases of the pandemic, but there is a need for measures that obtain information about variations in quality of life as the pandemic has been prolonged (Caycho-Rodríguez et al., 2022d). In this regard, the COV19 – Impact on Quality of Life (COV19-QoL; Repišti et al., 2020) which is comprised of six items that measure the impact of COVID-19 on people's quality of life. The COV19-QoL items were developed with the objective of trying to cover the main areas that may be affected by the pandemic, such as quality of life, mental health and personal safety (Singer et al., 2003; Zhang & Ma, 2020). In this sense, item 1 assesses the impact that the pandemic may have on overall quality of life. Items 2 and 3 assess how people perceive a deterioration in mental and physical health due to COVID-19. Items 4 and 5 assess changes in levels of anxiety and depression due to the pandemic, while item 6 assesses people's perception of their personal safety (Repišti et al., 2020).

The original psychometric study involved people from the general population and a clinical sample of patients with serious mental illness (Repišti et al., 2020). By means of an exploratory factor analysis (EFA), a single dimension was obtained that explained 64.13% and 58.88% of the variance in clinical and nonclinical samples, respectively. In addition, Cronbach's alpha coefficient values of α = 0.885 were obtained for clinical sample and α = 0.856 for nonclinical. The AFE used principal component analysis to assess the dimensionality of the COV19-QoL. However, the literature has pointed out that this procedure is the least recommended (Lloret-Segura et al., 2014) since, principal component analysis is a method of variable reduction and not one of factor analysis itself (Ferrando & Anguiano-Carrasco, 2010; Lloret-Segura et al., 2014). Principal component analysis considers both true and error variance, which leads to overestimation of factor loadings and thus biases the interpretation of the construct to be assessed (Timmerman & Lorenzo-Seva, 2011). A study on the adaptation of the COV19-QoL to Turkish culture also used the principal components method together with varimax rotation to evaluate the factor structure of the scale (Sümen & Adibelli, 2021). Additionally, the same study confirmed the unidimensionality of the scale by means of a confirmatory factor analysis (CFA) with adequate reliability indices. Another study that adapted the COV19-QoL to the Iranian language (Dehkordi et al., 2021) presented further evidence in favor of the unidimensional model, from the EFA and CFA with adequate reliability.

As can be seen, to date, all previous psychometric studies of the COV19-QoL have used procedures based on Classical Test Theory (CTT). However, for some years now, procedures based on Item Response Theory have been used to evaluate the psychometric properties of measurement instruments (Embretson & Reise, 2000). While the TCT provides a single score from the scores of each of the items that make up the scale, the IRT allows obtaining trait scores at the level of each item. Similarly, the TCT assumes and provides a single reliability value for all levels of the scores obtained, while the IRT provides reliability of each item at various levels of the trait. In addition, the psychometric properties based on the TCT are sample-dependent and, therefore, may vary between different samples. However, IRT assumes that psychometric properties are independent of the sample. Also, IRT provides item-level parameters, which would allow identifying those items that discriminate better and can measure traits more reliably. Thus, IRT would make it possible to obtain more valuable data on the psychometric properties of the measurement instruments and items of the COV19-QoL, as well as to guide procedures for their continuous improvement.

On the other hand, previous psychometric studies of the VOC19-QoL did not assess measurement invariance (MI). MI allows for evidence that the same underlying construct can be measured between different comparison groups. This would ensure that differences between groups can be interpreted as true differences in the underlying construct. If MI is not met, then the measurement is biased and between-group differences would not reflect true differences in the underlying trait. Therefore, the comparative results may not be valid and the conclusions erroneous (Meredith, 1993; Widaman & Reise, 1997).

As noted, the psychometric properties of the COV19-QoL have been evaluated in samples from European countries, such as Bosnia and Herzegovina, Montenegro, North Macedonia and Serbia (Repišti et al., 2020), Turquía (Sümen & Adibelli, 2021) and Irán (Dehkordi et al., 2021). However, as far as is known in the scientific literature, there are no studies that have evaluated the psychometric properties or used the COV19-QoL in Spanish, and even less in a group of older adults in a Latin American country. In this sense, the aim of the present study was to translate into Spanish and evaluate the psychometric evidence of the COV19-QoL in a sample of Peruvian older adults based on methods derived from TCT and IRT. Specifically, we evaluated the evidence of validity based on the internal structure of the COV19-QoL, its reliability by the internal consistency method, the characteristics of the items and their MI according to sex. Based on previous evidence, the Spanish COV19-QoL is expected to maintain a unidimensional structure and good reliability (Dehkordi et al., 2021; Repišti et al., 2020; Sümen & Adibelli, 2021). Although some psychometric characteristics of the COV19-QoL have not been evaluated from IRT, it would be expected to have adequate discrimination and difficulty parameters, as has been reported for other quality of life measures (Tomás et al., 2018; Vahedi, 2010). In addition, it has been suggested that there is a need for psychometrically robust and accurate indicators of quality of life that are useful for decision-making (Cella et al., 2002). Finally, COV19-QoL is expected to be invariant between groups of men and women, as other measures of quality of life in different samples (Chavez et al., 2018; Revell et al., 2009). A study of 33,019 older adults in low- and middle-income countries indicated that male older adults reported better quality of life than female older adults. However, another study reported that gender was not a significant predictor of quality of life in older adults during the pandemic (Bidzan-Bluma et al., 2020). Given this incongruence of findings, it is important to have a measure that allows us to have significant comparative results of quality of life between groups of different genders.

## Method

### Participants

The participants were 298 older adults (58.1% women, 41.9% men) with a mean age of 65.34 years (SD = 11.33). Inclusion criteria were: 1) age > 60 years and 2) consent to participate in the study. On the other hand, the exclusion criteria were: 1) age < 60 years; 2) not giving consent to participate in the study and, 3) not having access to the Internet to complete the survey. The vast majority of older adults surveyed had a permanent job (57.4%), followed by those unemployed (25.5%), with temporary jobs (13.8%) and retired (3.4%). Likewise, most of them have complete (30.5%) or incomplete (37.9%) primary education, followed by older adults with complete university studies (16.8%); while the rest of the participants (14.8%) reported having only complete or incomplete secondary education, incomplete university studies, as well as complete and incomplete technical studies. Regarding the time of exposure to information related to COVID-19, 43% indicated being exposed between 1 to 3 h, 32.2% were exposed between 3 to 5 h, 19.1% between 5 to 7 h, while 5.7% were exposed for more than 7 h. On the other hand, 70.1% indicated that they were widowed at the time of answering the survey, 19.1% were single, 6.7% were married, 1.7% were divorced and 2.3% were cohabiting.

### Instruments

COV19 – Impact on Quality of Life (COV19-QoL; Repišti et al., 2020). The COV19-QoL measures the impact of the COVID-19 pandemic on major areas of mental health-related quality of life. It is comprised of six items that have five-point Likert-type response options (1 = strongly disagree to 5 = strongly agree). Participants were asked to rate their feelings and thoughts for each of the items over the past seven days. The total score ranges from 6 to 30 points, where higher scores would indicate a greater impact of the COVID-19 pandemic on mental health.

The original English version of the COV19-QoL was translated into Spanish using the forward and backward translation method (Beaton et al., 2000). The direct translation from English to Spanish was performed by one of the bilingual authors of this study. Then, another bilingual author, who did not know the original English version, back-translated the Spanish version into English. No contradictions were reported between the original English version and the back-translated version. The researchers analyzed and discussed challenging phrases or ambiguous words in the final translated version until they reached an agreement on the appropriate translation. The translated version was preliminarily applied to 10 older adult participants who represented the target population. This procedure aimed to ensure cultural adaptability and to have information that the instructions and items were clearly expressed. This procedure did not report any problems in the Spanish translated version nor the need for changes. Table 1 shows the original English version and the Spanish version of the COV19-QoL.

**Table 1:** Original English version and Spanish version of the COV19-QoL

Original English version of the COV19-QoL	Spanish version of the COV19-QoL
Item 1: Due to the spread of the coronavirus, I think my quality of life is lower than before	Item 1: Debido a la propagación del coronavirus, creo que mi calidad de vida es más baja que antes
Item 2: Due to the spread of the coronavirus, I think my mental health has deteriorated	Item 2: Debido a la propagación del coronavirus, creo que mi salud mental se ha deteriorado
Item 3: Due to the spread of the coronavirus, I think my physical health may deteriorate	Item 3: Debido a la propagación del coronavirus, creo que mi salud física puede deteriorarse
Item 4: Due to the spread of the coronavirus, I feel more tense than before	Item 4: Debido a la propagación del coronavirus, me siento más tenso que antes
Item 5: Due to the spread of the coronavirus, I feel more depressed than before	Item 5: Debido a la propagación del coronavirus, me siento más deprimido que antes
Item 6: Due to the spread of the coronavirus,.I feel that my personal safety is at risk	Item 6: Debido a la propagación del coronavirus, siento que mi seguridad personal está en riesgo

### Procedure

The study followed the principles of the Declaration of Helsinki and obtained the approval of the Institutional Committee for the Protection of Human Subjects in Research of the University of Puerto Rico (No. 2223–006). An online questionnaire was developed using Google Form, consisting of sociodemographic questions and the COV19-QoL. The questionnaire was distributed online through snowball sampling to reach as many participants as possible who met the inclusion criteria (Atkinson & Flint, 2004). In this sense, older adults who met the inclusion criteria were identified and asked to suggest others who were interested in participating. Their telephone number was used to make the first contact. Once the older adult met the inclusion criteria and agreed to participate, he/she was asked for his/her e-mail address or, if not, the e-mail address of a close relative to send the survey online. It was explained to the older adults that their participation was voluntary and they were free to participate in the study or not and to stop answering at any time they wished. Older adults who agreed to participate in the study electronically confirmed their decision to volunteer and then began answering the questions. On average, answering the online questionnaire took about five minutes.

### Data análisis

The statistical analysis was performed with the RStudio environment (RStudio Team, 2018) for R (R Core Team, 2019). Specifically, the packages "lavaan" was used to perform the CFA model (Rosseel, 2012), "semTools" (Jorgensen et al., 2018) and "mirt" for the analyses based on the TRI model (Chalmers, 2012a, 2012b).

### Content validity evidence

Content validity was assessed based on the criteria of clarity (degree to which the item is clearly worded and understandable), coherence (degree of relationship between the item and the construct assessed) and relevance (degree to which the item is important and should be included to assess the perceived impact of COVID-19 on quality of life) of the COV19-QoL items. This evaluation was performed by 6 expert health professionals (psychologists and psychiatrists). The criteria of clarity, coherence and relevance were rated from 0 (not at all relevant/coherent/clear) to 3 (totally relevant/coherent/clear). The criteria were quantified using Aiken's V coefficient (Aiken, 1980) and its 95% confidence intervals (Penfield & Giacobbi, 2004). Values of V greater than 0.70 indicate a positive evaluation of the items at the sample level, while values of the lower limit of the confidence interval greater than 0.59 indicate a positive evaluation at the population level.

### Descriptive analysis of the ítems

Descriptive statistics were calculated for all items of the COV19-QoL, including mean (M), standard deviation (SD), skewness (g1), kurtosis (g2) and polychoric correlation matrix. Values of g1 and g2 between—1.5 and + 1.5 indicate an approximation of normal distribution of the data (Byrne & Campbell, 1999).

### Evidence of validity based on internal structure

A CFA is performed to assess whether the data fit the original theoretical model. The Diagonally Weighted Least Squares with Mean and Variance corrected (WLSMV) estimation method was used due to the ordinal nature of the VOC19-QoL items (Li, 2016). The goodness-of-fit of the model was evaluated using the chi-square index (χ2), comparative fit index (CFI), standardized root mean square residual (SRMR) and root mean square error of approximation (RMSEA) (Kline, 2015). The above indices were considered acceptable according to the following values: CFI > 0.95, RMSEA < 0.08 and SRMR < 0.08 (Kline, 2015; Schumacker & Lomax, 2015).

### Reliability

Reliability was assessed based on the internal consistency method using Cronbach's alpha (α; Cronbach, 1951) and McDonald's Omega (ω; McDonald, 1999) coefficients. Values of ω and α > 0.80 are adequate (Raykov & Hancock, 2005).

### Item calibration with the 2-PML Graduated Response Model (GRM)

After confirming the factor structure of the COV19-QoL, discrimination, difficulty and informativeness were evaluated based on IRT (Chalmers, 2012a, 2012b). This model allows more detailed information to be obtained at the item level compared to the TCT. For this purpose, an extension of the 2-parameter logistic model (2-PLM) designed for ordinal polytomous items was used (Hambleton et al., 2010). The IRT model was fitted with the C2 test for ordinal items (Cai & Monroe, 2013). In addition, the following fitting criteria were used: RMSEA ≤ 0.08, SMSR ≤ 0.05 (Maydeu-Olivares & Joe, 2014), CFI and TLI ≥ 0,95 (Cai et al., 2021; Lubbe & Schuster, 2019). Discrimination indices (a) and difficulty (b) were calculated. Values of a > 1.0 are considered highly discriminant (Baker, 1985). Four thresholds were estimated for parameter b, due to the presence of five response categories. The IRT provides information functions for each item and for all items together. In this sense, the Item Information Curves (IIC) and Test Information Curve (TIC) were also calculated. The IICs indicate the range of construct levels at which the items are best at discriminating between individuals. The items that present a higher slope in the CCI are the most differentiated and allow to distinguish adequately between different degrees of the evaluated construct, which makes them more sensitive to changes (Hambleton et al., 1991). The TIC indicates the amount of information contained in a test at all points at different points on the latent trait. Higher values of information at a specific trait level indicate that a test provides more information and is more accurate about an individual's latent trait at that level. That is, more information would reflect greater measurement precision at different levels of the construct being measured (Embretson & Reise, 2000).

### Measurement Invariance (MI)

MI was evaluated using the multigroup CFA method where different nested models were compared. Specifically, two models, a restricted model and an unrestricted model, considered baseline, are compared to observe the presence or absence of significant differences in model fit. If the fit of the restricted model is not significantly worsened compared to the baseline model, then the presence of invariance of the evaluated parameters is accepted (Dimitrov, 2010a, 2010b). Three invariance models were evaluated: configurational invariance model, where the presence of the same factor structure between groups is evaluated without restrictions (baseline model); metric invariance model, where factor loadings are restricted to equality between groups and; scalar invariance model, where factor loadings and intersections are similarly restricted in the compared groups. Structural invariance was examined by constraining latent means to be equal between sex groups (Dimitrov, 2010a, 2010b). A nonsignificant χ2 (Δχ2) variance value is considered to indicate the presence of MI between groups. However, the Δχ2 is too strict and sensitive to differences in sample size (Dimitrov, 2010a, 2010b; Putnick & Bornstein, 2016). Therefore, the ΔCFI was the main criterion for comparing the more constrained models with the less constrained models (Putnick & Bornstein, 2016).

## Results

Table 2 shows that the five items of the COV19-QoL are clear, relevant and consistent, both at the sample level (V > 0.70) and at the population level (Li > 0.59). Table 3 shows the mean, standard deviation, skewness and kurtosis of all items of the Spanish version of the COV19-QoL, as well as the polychoric correlation matrix. Item 3 ("Because of the spread of the coronavirus, I think my physical health may deteriorate") has the highest average score; whereas, item 5 ("Because of the spread of the coronavirus, I feel more depressed than before") has the lowest average score. It is observed that the six items are normally distributed, because the skewness and kurtosis values are within the range—1.5 to 1.5. The presence of a floor effect is observed, that is, more than 15% of the participants obtain the minimum possible score on an item (Terwee et al., 2007). Likewise, the correlation matrix allows us to observe moderate relationships between the items.

**Table 2:** Aiken's V for assessing the clarity, coherence, and relevance of MFS items

Item	Clarity (*n* = 6)				Coherence (*n* = 6)				Relevance (*n *= 6)			
	M	SD	V	IC95%	M	SD	V	IC95%	M	SD	V	IC95%
Item 1	4.00	.00	1.00	.90–1.00	3.00	1.10	.75	.58-.87	4.00	.00	1.00	.90–1.00
Item 2	4.00	.00	1.00	.90–1.00	3.67	.52	.92	.78-.97	3.33	.82	.83	.68-.92
Item 3	3.83	.41	.96	.83-.99	3.67	.52	.92	.78-.97	3.50	.84	.88	.72-.95
Item 4	4.00	.00	1.00	.90–1.00	3.67	.52	.92	.78-.97	3.50	.84	.88	.72-.95
Item 5	3.50	.84	.88	.72-.95	3.33	.82	.83	.68-.92	3.00	1.10	.75	.58-.87
Item 6	3.33	.82	.83	.68-.92	3.50	.84	.88	.72-.95	3.83	.41	.96	.83-.99

*M* Mean, *SD* Standard deviation, *V* Aiken's V coefficient, IC95% = 95% confidence intervals

**Table 3:** Descriptive analysis of the items

Ítem	Opciones de respuesta					Estadísticos descriptivos				Matriz de correlaciones					
	**1**	**2**	**3**	**4**	**5**	**M**	**SD**	**g**^**1**^	**g**^**2**^	**I1**	**I2**	**I3**	**I4**	**I5**	**I6**
I1	26.8%	20.1%	25.8%	17.1%	10.1%	2.63	1.31	0.25	-1.08	-					
I2	32.6%	20.5%	26.8%	13.4%	6.7%	2.41	1.25	0.42	-0.89	0.67	-				
I3	24.5%	15.8%	29.2%	21.5%	9.1%	2.75	1.29	0.04	-1.11	0.65	0.69	-			
I4	26.2%	20.5%	30.9%	16.1%	6.4%	2.56	1.22	0.22	-0.93	0.63	0.75	0.70	-		
I5	36.2%	22.8%	17.8%	15.4%	7.7%	2.36	1.32	0.55	-0.95	0.56	0.77	0.59	0.73	-	
I6	30.2%	19.8%	23.5%	18.1%	8.4%	2.55	1.31	0.29	-1.11	0.58	0.70	0.62	0.74	0.74	-

*M* Mean, *SD* Standard deviation, g1 Skewness, g2 Kurtosis, I1 Item 1, I2 Item 2, I3 Item 3, I4 Item 4, I5 Item 5, I6 Item 6

**Fig. 1:**
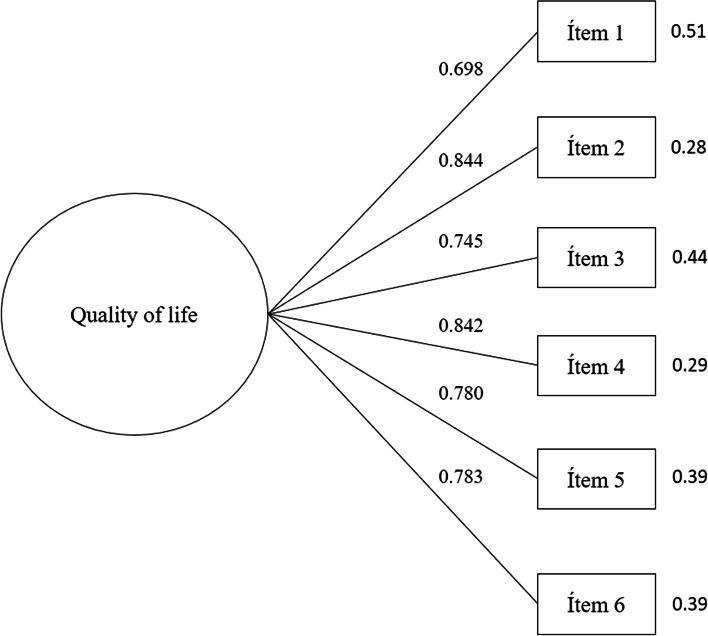
Confirmatory Factor Analysis of the scale

The undimensional factorial model described by Repišti, et al. (2020), Sümen y Adibelli (2021) y Dehkordi et al. (2021) was used to evaluate the goodness of fit. The CFA indicated an excellent fit for the unidimensional model in the total sample (χ2 = 19.08; df = 9, χ2/df = 2.12, CFI = 0.985, TLI = 0.974, SRMR = 0.032, RMSEA = 0.064, 90% CI [0.03, 0.10]). The factor loadings of the COV19-QoL were significant and ranged from 0.698 to 0.844. Therefore, the unidimensional construct of quality of life was confirmed and is shown in Fig. 1. Cronbach's alpha (α = 0.903) and Omega (ω = 0.905) coefficients indicated excellent internal consistency reliability.

**Fig. 2:**
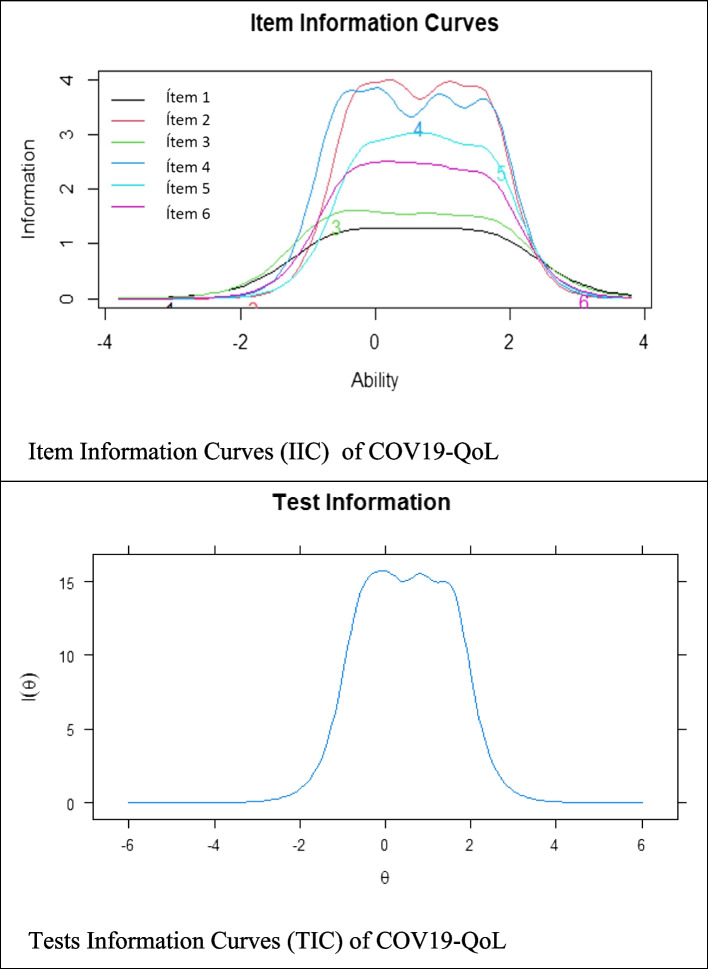
Item and Test Information Curves for the COV19-QoL

IRT was used to evaluate the characteristics of the VOC19-QoL items. The IRT results are presented in Table 4 and Fig. 2. All values of parameter a were greater than 1.0; while, with respect to the difficulty parameters, all threshold values increased monotonically. Therefore, the IRT-based results indicated that the items of the Spanish version of the COV19-QoL have an appropriate difficulty and discriminant capacity. In addition, the estimated GRM model has adequate fit indices. The item information curve (IIC) and the test information curve (TIC) (Fig. 2) show that items 2 and 4 are more accurate in assessing quality of life. In addition, the TIC shows that the factor is more reliable (accurate) in the range of the scale between -1 and 2.

**Table 4:** Parameters of the GRM model items

Item	*a*	b_1_	b_2_	b_3_	b_4_	C^2^	RMSEA	SRMR	TLI	CFI
I1	2.061	-0.747	-0.008	0.836	1.654	37.47935	0.1032	0.058	0.97	0.98
I2	3.665	-0.442	0.161	0.900	1.590					
I3	2.333	-0.839	-0.228	0.677	1.610					
I4	3.704	-0.661	-0.027	0.803	1.566					
I5	3.087	-0.358	0.290	0.832	1.566					
I6	2.849	-0.545	0.085	0.744	1.542					

I1 Item 1, I2 Item 2, I3 Item 3, I4 Item 4, I5 Item 5, I6 Item 6, a Discrimination index, b Difficulty index, C^2^ Test for ordinal items, *RMSEA* Root mean square error of approximation, *SRMR* Standardized root mean square residual, *TLI* Tucker–Lewis index, *CFI* Comparative fit index

When evaluating the MI of the COV19-QoL, the multigroup CFA indicated that the configurational model has good fit indices. Likewise, both the metric and scalar invariance model presented a ΔCFI less than 0.01. Overall, the results suggest evidence of gender-dependent MI of the unidimensional COV19-QoL due to consistent fit indices despite model constraints s increasing levels of model constraint. The evidence of scalar invariance allowed us to compare the means of the factor (Byrne et al., 1989; Steenkamp y& Baumgartner, 1998). Therefore, the equal of the means of the factor was evaluated. The change of ΔCFI indicated the preference for the equal means model (Table 5). Possible differences between sex groups were explored. Using the sample of men as a reference group, the mean factor for the perception of the impact of COVID-19 on quality of life was higher in the group of women (difference =  − 1.72, SE = 0,31).

**Table 5:** COV19-QoL invariance indices according to sex

	χ^2^	df	p	CFI	RMSEA	Δx^2^	ΔdF	ΔCFI	ΔRMSEA
M1	34.940	18	< .01	0.981	0.163	-	-	-	-
M2	42.443	23	< .01	0.980	0.148	6.9725	5	0.001	0.015
M3	59.566	40	< .01	0.983	0.104	18.8998	17	0.003	0.044
M4	118.820	41	< .01	0.979	0.114	6.7946	1	0.004	0.011

M1 Invariancia configuracional, M2 Invarianza métrica, M3 Invarianza escalar, M4 Equal means, χ2 Chi square, df degrees of freedom, CFI Comparative Fit Index, RMSEA Root Mean Square Error of Approximation, Δχ2 Differences in Chi square, Δdf Differences in degrees of freedom, ΔRMSEA Change in Root Mean Square Error of Approximation; ΔCFI Change in Comparative Fix Index

## Discussion

The aim of the present study was to translate into Spanish and evaluate the psychometric evidence of the COV19-QoL in a sample of Peruvian older adults based on methods derived from TCT and IRT. The evaluation of the content validity of the COV19-QoL items indicated that all are sufficiently relevant, coherent and clear to adequately represent the perception of the impact of COVID-19 on quality of life. Therefore, the contents of the six items are adequate to be applicable to the sample of Peruvian older adults. The results of the CFA confirmed that the Spanish version of the COV19-QoL has a similar undimensional structure to the original versión (Repišti et al., 2020) and validations in Turkey (Sümen & Adibelli, 2021) and Irán (Dehkordi et al., 2021). Therefore, deterioration in general quality of life, mental and physical health, changes in anxiety and depression levels, as well as perception of personal safety were shown to be adequate indicators of the impact of the COVID-19 pandemic on the quality of life of older adults. The original COV19-QoL was validated in general population and clinical sample of patients with some serious mental illness (Repišti et al., 2020), the Turkish adaptation and validation was performed in general population diagnosed or not with COVID-19 (Sümen & Adibelli, 2021); while, the Iranian version was validated in general population (Dehkordi et al., 2021). However, the present study added new evidence on the factorial structure of the COV19-QoL in a sample of older adults. Even so, further studies should be conducted to evaluate the psychometric properties of the scale, especially in different clinical and non-clinical samples of older adults from different regions of Peru and other Latin American countries to further clarify the factorial structure of the COV19-QoL. Likewise, the present study showed that the unidimensional model of the COV19-QoL had a very good reliability by the internal consistency method. Both the alpha and omega coefficient values were above 0.80. These results were in line with those reported in the original study and the Turkish and Iranian versions (Dehkordi et al., 2021; Repišti et al., 2020; Sümen & Adibelli, 2021). Therefore, the COV19-QoL is an accurate measure of the impact of the pandemic on the quality of life of older Peruvian adults.

Similarly, IRT was used to evaluate the characteristics of the COV19-QoL items. The discrimination parameters of all COV19-QoL items were greater than 1, thus, they can adequately discriminate between low, medium and high levels of the impact of the COVID-19 pandemic on quality of life. Items 2 and 4 are the most discriminative. Item 2 is associated with the deterioration of mental health as a result of the pandemic. It has been suggested that older adults experienced greater symptoms of depression, anxiety, stress, ageism and loneliness during the pandemic compared to pre-pandemic data (Lebrasseur et al., 2021; Parlapani et al., 2021). However, older age may also be an important factor in buffering the impact of COVID-19 on mental health (Parlapani et al., 2021). Item 4 refers to a greater degree of mental stress during the pandemic. This is something reported previously, where being exposed to information about COVID-19 infections and deaths generated greater stress in older adults, which exacerbated their fears (Portacolone et al., 2021). Therefore, these items could be useful in identifying whether an older adult is at risk for a greater impact of the pandemic on his or her quality of life. On the other hand, the monotonic increase in the difficulty parameters would indicate that, a greater perceived impact of the pandemic on quality of life is necessary to answer the higher response options of the VOC19-QoL. In addition, the CIT indicated a relatively high degree of measurement accuracy when the degree of impact of the pandemic on quality of life was medium to high. Therefore, IRT analysis indicated that the six items of the COV19-QoL have adequate characteristics for assessing the impact of the pandemic on the quality of life of older adults.

When testing the gender-dependent MI of the COV19-QoL using a multigroup CFA, the three levels of restriction provided support for the invariance of the factor structure. Therefore, COV19-QoL performs equivalently for males and females. Specifically, the presence of configural invariance indicated that, the impact of the COVID-19 pandemic on quality of life, as assessed by the COV19-QoL, has equal significance in men and women. The presence of metric invariance suggested that, items on the COV19-QoL are interpreted and responded to similarly between groups. As such, some variation in the impact of the COVID-19 pandemic on quality of life would cause the same variation in COV19-QoL scores in the women and men assessed. Evidence of scalar invariance would indicate that the relationship between observed and latent COV19-QoL scores is invariant between groups. No previous studies have evaluated the gender-specific MI of COV19-QoL. Therefore, we provide an empirical basis for valid comparisons for future studies assessing gender differences in the impact of the COVID-19 pandemic on quality of life. This is possible, as the VOC19-QoL scores have the same significance among older adults of both genders.

Once the invariance was tested, the latent means of the perception of the impact of COVID-19 on quality of life were compared between older adult men and women. The results show differences between men and women, with the latter perceiving a greater impact of COVID-19 on their quality of life, but these differences were small. This finding is related to previous studies that reported that the COVID-19 pandemic had a negative psychological impact on women compared to men (Broche-Pérez et al., 2022; Wang et al., 2020). However, a previous study reported the opposite result, where no difference was observed in the impact of COVID-19 emergency status on the quality of life of older Peruvian adults of both sexes (Tenorio-Mucha et al., 2021). In this sense, the present finding and the previous results give evidence that the relationship between quality of life and gender are not entirely conclusive. The greater impact perceived by women could be associated with the fact that this group tends to show greater reactivity in the neural networks related to mental health problems in the face of an infectious disease (Liu et al., 2020). Gender roles could also explain this result. For example, during the pandemic, women were more likely to care for other family members than men, which may have led to a lower perception of their quality of life due to the excessive burden on their households (López-Ruiz et al., 2022). It has been suggested that this may have exacerbated pre-pandemic gender differences (Bau et al., 2022). Likewise, the measures taken by the Peruvian government during the pandemic were so strict that even adults over 65 years of age were prevented from taking walks near their homes and were denied the possibility of physical activity, which is so necessary for their well-being (Tenorio-Mucha et al., 2021). Although the restrictions were relaxed at the time and priority was given to vaccinating adults over 60 years of age, the restrictions may have had a negative impact on people's perception of their quality of life.

The study has limitations that should be considered when interpreting the findings. First, non-probability snowball sampling was used, which meant that the sample is not representative of the general population of older adults in Peru. In addition, the non-probabilistic sampling generated a very heterogeneous sample, where the majority reported having a permanent job, complete or incomplete primary education and being widowed. This made it difficult to generalize the results to the general population of older adults. Second, the use of an online survey to collect information limited the participation of those older adults who did not have access to the Internet or experience with this type of online survey. Future studies should test whether the present findings are reproducible in a more representative sample. Third, information on diagnoses of mental health problems was not obtained. Therefore, the sensitivity and specificity of the scale were not assessed. However, this should be considered in future studies. Fourth, due to the cross-sectional design, the stability of the COV19-QoL over time could not be assessed, that´s why future studies with longitudinal designs are needed. Fifth, the use of a self-report measure of quality of life could generate problems of social desirability bias. However, it is possible to think that this type of bias has little influence on the results of the present study, since anonymity was guaranteed in the collection of information. Sixth, as mentioned above, soil effects on COV19-QoL were reported. Therefore, it is possible that the older adults who participated in the present study perceived a lower impact of COVID-19 on their quality of life compared with comparable groups of older adults in other countries who have been more affected by COVID-19.

## Conclusion

As we know, the pandemic has generated problems in people's mental health and quality of life. Therefore, it is a priority to gather information on the impact of the COVID-19 pandemic on the quality of life of specifically vulnerable groups, such as older adults. Therefore, the results allow us to consider the COV19-QoL as an instrument with adequate psychometric properties to identify people with greater vulnerability in their quality of life as a consequence of the pandemic. Specifically, the COV19-QoL has a unifactorial structure, high reliability by the internal consistency method and measurement invariance by gender. In addition, all items presented adequate discrimination and difficulty indices. Thus, the items allow adequate discrimination between low, medium and high levels of the impact of the COVID-19 pandemic on quality of life. Likewise, it is necessary to perceive a greater impact of the pandemic on quality of life to respond to the higher response options of the COV19-QoL. In this way, having a valid measure of the impact of the pandemic on quality of life will help health professionals to obtain information on the main areas affected by the pandemic (general quality of life, deterioration of mental and physical health, changes in anxiety and depression levels, and personal safety) and thus develop appropriate interventions to mitigate the impact of the pandemic on the quality of life of older adults. To mitigate the impact of the pandemic, strategies for stress management, anxiety, depression, improvement of physical health and personal safety can be provided through technological support such as telecounseling and telemedicine (Ornell et al., 2020; Zhou et al., 2020). Finally, the study provides a measure in addition to other scales recently adapted and validated in Peru to assess aspects of mental health in the older adult population during the COVID-19 pandemic, such as the Fear of COVID-19 Scale (Caycho-Rodríguez et al., 2022a) or the Coronavirus Anxiety Scale (Caycho-Rodríguez et al., 2022c).

## Data Availability

All data related to this study are available from the authors upon request. The data are not yet publicly available because the project group is still processing it.

## Abbreviations

2-PLM: 2-Parameter logistic model
BBQ: Brunnsviken brief quality
CFA: Confirmatory factor analysis
CFI: Comparative fit index
COV19-QoL: COV19 – Impact on Quality of Life
CTT: Classical Test Theory
EFA: Exploratory factor analysis
g1: Skewness
g2: Kurtosis
GRM: Graduated Response Model
IIC: Item Information Curves
INEI: Instituto Nacional de Estadística e Informática
IRT: Item Response Theory
M: Mean
MI: Measurement invariance
MINSA: Ministerio de Salud
MQLI: Multicultural Quality of Life Index
RMSEA: Root mean square error of approximation
SD: Standard deviation
SRMR: Standardized root mean square residual
TIC: Test Information Curve
WHO: World Health Organization
WHOQOL-BREF: World Health Organization Quality of Life-brief version
χ2: Chi-square
